# Determining the degradation efficiency and mechanisms of ethyl violet using HPLC-PDA-ESI-MS and GC-MS

**DOI:** 10.1186/1752-153X-6-63

**Published:** 2012-06-30

**Authors:** Wen-Hsin Chung, Chung-Shin Lu, Wan-Yu Lin, Jian-Xun Wang, Chia-Wei Wu, Chiing-Chang Chen

**Affiliations:** 1 Department of Science Application and Dissemination, National Taichung University of Education, Taichung 403, Taiwan, Republic of China; 2 Department of Plant Pathology, National Chung Hsing University, Taichung, 402, Taiwan, Republic of China; 3 Department of General Education, National Taichung, University of Science and Technology, Taichung, 403, Taiwan, Republic of China

**Keywords:** HPLC-PDA-ESI-MS, GC-MS, EV dye, Zinc foil, Degradation mechanism

## Abstract

**Background:**

The discharge of wastewater that contains high concentrations of reactive dyes is a well-known problem associated with dyestuff activities. In recent years, semiconductor photocatalysis has become more and more attractive and important since it has a great potential to contribute to such environmental problems. One of the most important aspects of environmental photocatalysis is in the selection of semiconductor materials like ZnO and TiO_2_, which are close to being two of the ideal photocatalysts in several respects. For example, they are relatively inexpensive, and they provide photo-generated holes with high oxidizing power due to their wide band gap energy. In this work, nanostructural ZnO film on the Zn foil of the Alkaline-Manganese Dioxide-Zinc Cell was fabricated to degrade EV dye. The major innovation of this paper is to obtain the degradation mechanism of ethyl violet dyes resulting from the HPLC-PDA-ESI-MS analyses.

**Results:**

The fabrication of ZnO nanostructures on zinc foils with a simple solution-based corrosion strategy and the synthesis, characterization, application, and implication of Zn would be reported in this study. Other objectives of this research are to identify the reaction intermediates and to understand the detailed degradation mechanism of EV dye, as model compound of triphenylmethane dye, with active Zn metal, by HPLC-ESI-MS and GC-MS.

**Conclusions:**

ZnO nanostructure/Zn-foils had an excellent potential for future applications on the photocatalytic degradation of the organic dye in the environmental remediation. The intermediates of the degradation process were separated and characterized by the HPLC-PDA-ESI-MS and GC-MS, and twenty-six intermediates were characterized in this study. Based on the variation of the amount of intermediates, possible degradation pathways for the decolorization of dyes are also proposed and discussed.

## Background

It is estimated that over 700,000 tons of dyes and pigments are produced annually worldwide, 20% of which are utilized for textile dyeing and finishing processes [1]. Many of these synthetic dyestuffs cannot be removed using conventional treatments due to their complex polyaromatic structures, resulting in various environmental problems [2]. The textile, paper, food, cosmetic, and leather goods industries are all major consumers of triphenylmethane dyes [1,2]. Previous reports [3,4] have demonstrated the photodegradation of triphenylmethane dyes containing *N*-alkylamine groups via consecutive N-de-alkylation reactions. Other studies have reported that thyroid peroxidase-catalyzed oxidation of triphenylmethane dyes could result in the formation of various *N*-de-alkylated primary and secondary aromatic amines, with structures similar to those of aromatic amine carcinogens [5]. Previous studies [6,7] on the photocatalytic degradation of nitrogen-containing aromatic compounds have demonstrated that both electrons and hydroxyl radicals transform amine functional groups.

Zinc oxide is an important solid state material possessing photocatalytic [3] and piezoelectric properties, as well as demonstrating field emission and lasing action with a wide range of potential technological applications [8,9]. Recently, a variety of methods have been developed for the synthesis of nanostructural ZnO, including hydrothermal, vapor-liquid–solid, vapor solid, and other solution processes [10-21]. A low-temperature chemical-liquid deposition method has been employed to grow oriented ZnO nanorods by continuously supplying Zn ions from a Zn foil to form a ZnO thin film in aqueous formaldehyde solution [22]. Similar reactions have been achieved using Zn^2+^ salt with ethanol in the presence of amine to produce one-dimensional nanostructures of ZnO [23]. Hydrothermal reactions have also been used in the preparation of the ZnO nanorods, employing zinc acetate dissolved in ethanol with polyvinylpyrolidone and NaOH [24]. Heating zinc nitrate and NaOH in a mixture of ethylenediamine and water at 180°C for 20 h produces ZnO nanorods [25]. In the presence of ethylenediamine, the reaction of Zn foil with water under hydrothermal conditions (150-230°C) has reportedly yielded ZnO nanorods [26-31].

It has recently been discovered that cleaving a C-O single bond of the aliphatic alcohols on zinc metal surfaces produces ZnO nanoparticles [32]. Unfortunately, these techniques often require high temperatures. In addition, the reaction of Zn metal with liquid water may also produce ZnO nanostructures in a reaction associated with the evolution of hydrogen in acidic conditions [33]. The methods described in the literature generally use amines and other additives or zinc compounds at higher temperatures.

This study selected zinc foil obtained from waste Alkaline-manganese Dioxide-zinc cells as the substrate for the generation of ZnO nanostructures because the lattice matching between ZnO and Zn crystals facilitates the generation of ZnO nanostructures, and the zinc foil in these cells is waste material useful in the treatment of organic wastewater through photocatalysis. Zinc foil can serve as both reactant and substrate to support ZnO nanostructures without an additional substrate. The method is simple and practical, requiring only zinc foil, and may be performed at low temperatures. This simple method has not been previously reported in any studies. This makes it a suitable and economical approach to the treatment of organic wastewater. This study reports on the fabrication of ZnO nanostructures on zinc foil using a simple solution-based corrosion strategy, and provides detailed descriptions related to the synthesis, characterization, application, and implications of using Zn in this manner. Other objectives of this research include the identification of reaction intermediates to understand the underlying mechanisms in the degradation of EV dye as a model compound of triphenylmethane dye, with active Zn metal, using HPLC-ESI-MS and GC-MS. It is hoped that the results will provide a foundation for future environmental applications.

## Experimental

### Materials and reagents

**Figure 1:**
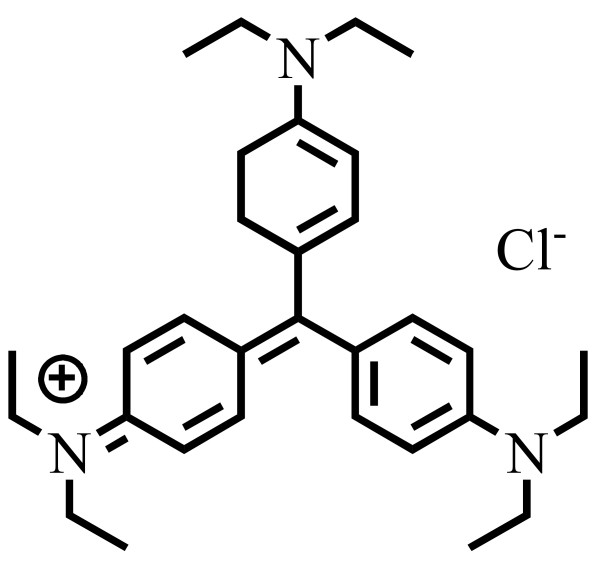
Chemical structure of EV.

Zn foils with 99.9% purity were 0.15 m in length, 0.15 m in width, and 2.5 × 10^-4^ m in thickness. Alkaline-Manganese Dioxide-Zinc cells was obtained from Eveready, Toshiba, and Panasonic. Ethyl violet dye was obtained from Tokyo Kasei Kogyo Co. The chemical structure of EV is shown in Figure 1. 4-Aminophenol (AP; analytical standard) was purchased from Riedel-deHaen. Reagent-grade ammonium acetate, nitric acid, sodium hydroxide, hydrogen chloride, and HPLC-grade methanol and acetone were purchased from Merck. All of the above agents were used as received without further purification.

### Degradation experiments

The Zn foils were ultrasonically washed in HPLC-grade acetone three times prior to use. A mixture solution was prepared by adding Zn foil (0.05 m × 0.05 m) to a 0.25 L aqueous solution containing EV at appropriate concentrations. The initial pH of the solution was adjusted by adding either NaOH or HNO_3_ solution to produce reactions of various pH values. At set intervals during the reaction, the solution was sampled. The residual dye and organic intermediates were analyzed using HPLC-PDA-ESI-MS and GC-MS. Dark experiments performed in a beaker with Zn foil also demonstrated the decolorization of the dye solution. Irradiation experiments were carried out for comparison using 15 W lamps to determine the stability of EV dye under UV or visible light irradiation. The 0.01 gL^-1^ EV solutions did not show significant de-coloration under UV irradiation without Zn foil. Following the reaction, the Zn foil was removed, washed with de-ionized water and ethanol several times, and then dried with nitrogen. These Zn foils were characterized using X-ray diffraction (XRD), field emission scanning electron microscopy (FE-SEM), and high resolution X-ray photoelectron spectrometry (HRXPS).

### Instruments and analytical methods

XRD patterns were recorded on a MAC Science, MXP18 X-ray diffractometer with Cu α radiation, operating at 40 kV and 0.08 A. FE-SEM measurement was carried out using a field-emission microscope (JEOL JSM-7401 F) operating at an acceleration voltage of 1.5 × 10^4^ V. HRXPS measurement was carried out with ULVAC-PHI XPS: PHI Quantera SXM to measure changes in the surface structure following reflux treatment. The binding energy values reported in the present work were corrected with a C1s peak at 284.8 eV to take into account charging effects.

A Waters ZQ LC/MS system equipped with Waters 1525 Binary HPLC pumps, a Waters 2998 Photodiode Array Detector, a Waters 717 plus auto sampler, and a Waters micromass-ZQ 2000 detector were used. The analysis of organic intermediates was accomplished using HPLC-PDA-ESI-MS following the readjustment of chromatographic conditions to make the mobile phase compatible with the working conditions of the mass spectrometer. Two types of eluent were employed in this study: solvent A, 0.025 M aqueous ammonium acetate buffer (pH 6.9); and solvent B, methanol. LC was carried out on an Atlantis ^TM^ dC18 column (0.25 m × 0.046 m i.d., 5 × 10^-6^ m film thickness). The flow rate of the mobile phase was set at 0.001 L.min^-1^. Column effluent was introduced into the ESI source of the mass spectrometer.

Solid-Phase extraction (SPE) was employed for the pre-concentration of irradiated samples prior to GC-MS analysis. GC/MS analysis was performed on a Perkin-Elmer AutoSystem-XL gas chromatograph interfaced with a TurboMass selective mass detector. Separation was carried out in a DB-5 capillary column (5% diphenyl/95% dimethyl-siloxane) with 60 m, 2.5 × 10^-4^ m i.d., and film thickness of 1.0 × 10^-6^ m. Electron impact (EI) mass spectra were monitored from 35 to 300 m/z. The ion source and inlet line temperatures were set at 220 and 280°C, respectively.

## Results and discussion

### FE-SEM-EDS

**Figure 2:**
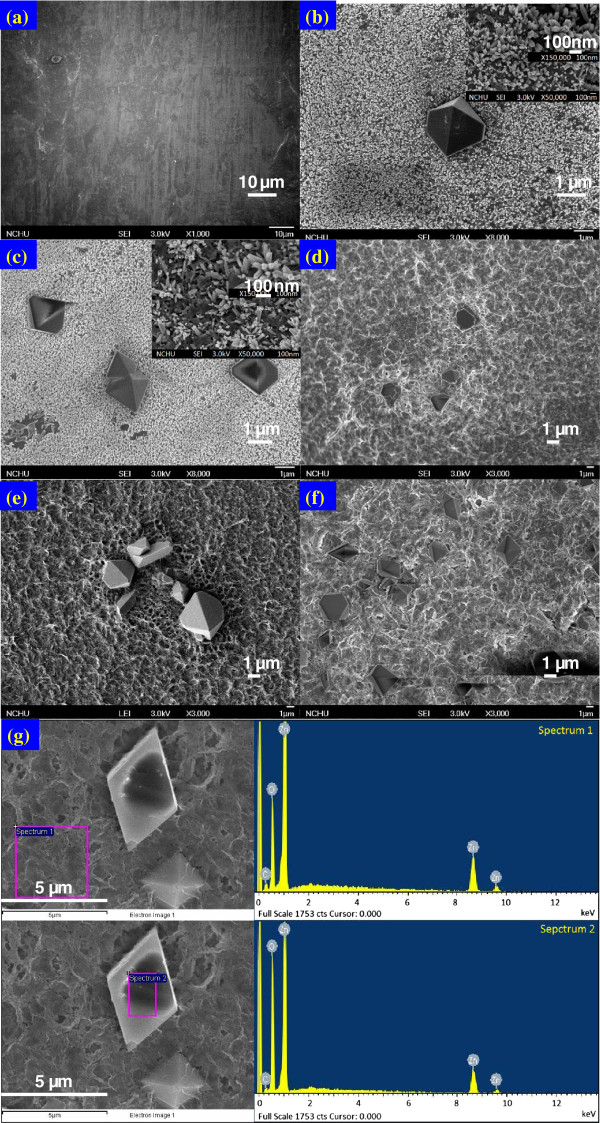
**FE-SEM images of Zn foil in DI water at room temperature for (a) 0 h, (b) 8 h, (c) 16 h, (d) 24 h, (e) 32 h, (f) 40 h.** Inset images are enlarged images for clarity. (**g**) Showed the EDX of various spot of the Zn surface after 24 h DI water exposure.

**Figure 3:**
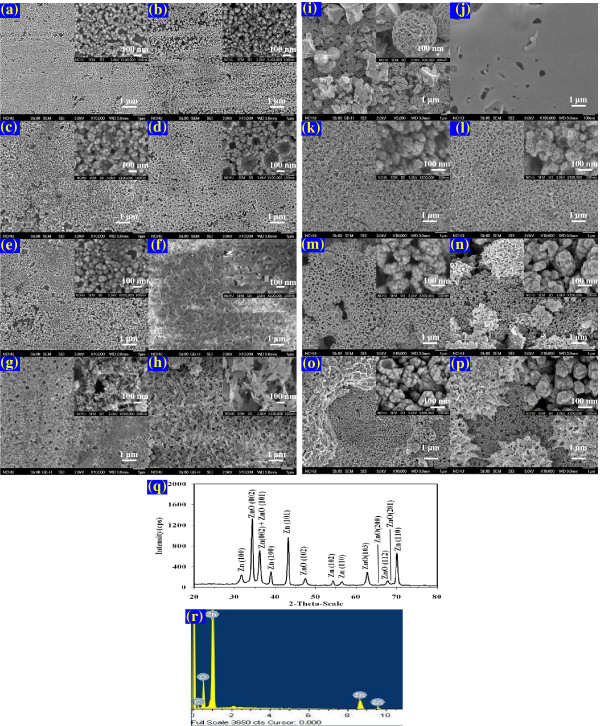
**FE-SEM images of Zn foil surface in EV solution (10 ppm, pH = 6) at room temperature for (a) 1 h, (b) 2 h, (c) 4 h, (d) 8 h, (e) 16 h, (f) 24 h, (g) 32 h, (h) 48 h, (i) 64 h, (j) 0 h, (k) 4 h, (l) 8 h, (m) 16 h, (n) 24 h, (o) 24 h under UV, (p) 24 h under Vis, (q) XRD pattern and (r) EDX of the surface powders.** (**a**)~(**i**) were obtained from pure Zn foil, (**j**)~(**r**) were obtained from Zn foil of Alkaline-Manganese Dioxide-Zinc Cell. Inset images are enlarged images for clarity.

Figures 2 and 3 show SEM images of the microcrystalline ZnO nanostructures generated on the surface of the Zn foil. The diameter of ZnO formations increased with reaction time. Figure 2 shows a top-down image of rods (polycrystallines) approximately 0.2-1 × 10^-7^ m in diameter and 1.5-3 × 10^-7^ m in length with microcrystalline structures approximately 3-5 × 10^-6^ m in diameter. The composition of the rods was characterized using energy dispersive spectroscopy (EDS), which revealed Zn and O as the only elementary components with an oxygen deficiency (Zn:O ~ 2:1 atomic ratio) [34]. Figure 3 shows the growth of ZnO film on a variety of Zn foils.

### XRD

**Figure 4:**
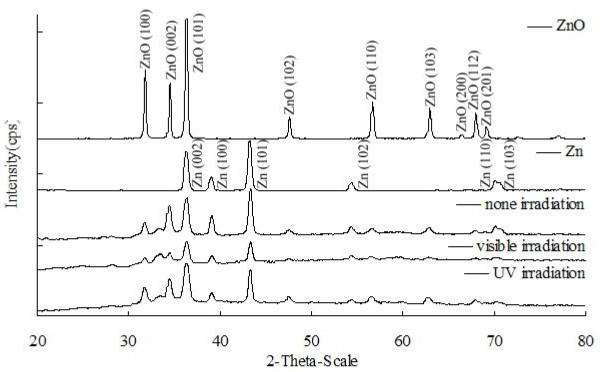
The XRD patterns of ZnO/Zn film in DI water under UV and visible irradiation for 64 h.

Figure 4 shows the XRD pattern of ZnO samples prepared in DI water at room temperature without/with irradiation. All absorption peaks corresponded well with ZnO (JCPDS card No. 36-1451) and Zn (JCPDS card No. 4-831). No obvious diffraction patterns were observed for impurities. The characteristic diffraction pattern of ZnO (1 0 0), (0 0 2), and (1 0 1), together with the diffraction pattern of Zn (0 0 2), (1 0 0), and (1 0 1) appeared in all samples.

### XPS

**Figure 5:**
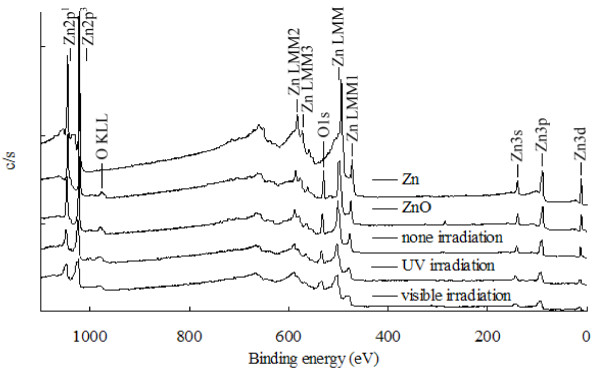
The XPS spectra of ZnO/Zn film in DI water under UV and visible irradiation for 64 h.

Figure 5 shows the XPS spectra of the ZnO samples obtained under various conditions. The binding energies of O1s in all samples were within 529.1-531.1 eV, which further confirmed the presence of Zn^2+^ and O^2-^ in all samples.

From the results of analysis, the natural oxidation of zinc metal by oxygen dissolved in water is rather slow due to the surface oxide layer at room temperature. However, with dye solutions, this spontaneous oxidation reaction can be drastically accelerated, enabling the rapid generation of ZnO nano-clusters on the surface of the zinc foil. The reaction between Zn and O_2_ in an aqueous solution is generally recognized to produce ZnO, as shown in Equations (1, 2, 3 and 4).

(1) $$\text{Zn}\to {\text{Zn}}^{2+}+{2e}^{-}E{}^{\circ}=0.76V$$

(2) $$1/{2O}_{2}+H_{2}O+{2e}^{-}\to {2\text{OH}}^{-}\;E{}^{\circ}=0.80V$$

(3) $${\text{Zn}}^{2+}+{2\text{OH}}^{-}\to \text{ZnO}+H_{2}O\;E{}^{\circ}=0.49V$$

(4) $$\text{Zn}+\;1/{2O}_{2}\to \text{ZnO E}{}^{\circ}=\;2.05V$$

The results obtained by reacting Zn metal with water are encouraging and have led to an examination of the reaction of ethyl violet dye in aqueous solutions. This study describes a very simple method to generate ZnO nanostructures in which ethyl violet dye is decomposed through a reaction of liquid water with metals. Similar reaction of Zn metal with liquid water may also produce ZnO nanostructures in a reaction associated with the evolution of hydrogen in acidic conditions. The methods described in the literature generally use amines and other additives or zinc compounds at higher temperatures [33].

### Photoirradiation experiments

**Figure 6:**
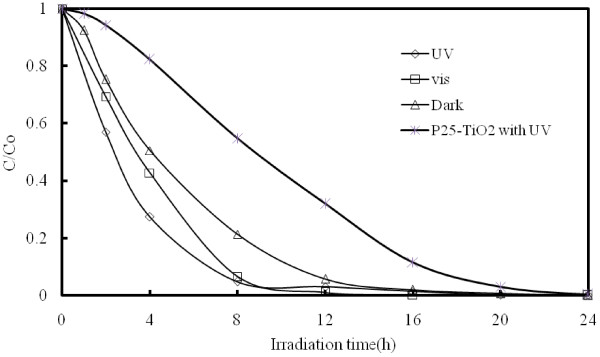
**The direct degradation of EV at dark and different wavelength irradiation: 365 nm and visible light.** (EV = 10 ppm, pH = 6, Zn foil = 5 × 5 cm^2^).

Figure 6 presents typical kinetic data illustrating the effects of photo-irradiation. The experiment with Zn in completely dark conditions shows that approximately 80% of the EV dye on the Zn surface had degraded after 8 hr. The results of the photo-irradiation experiments demonstrate that under visible light conditions, the EV concentration decreased by 93% after 8 hr of irradiation, while nearly 96% of the EV was removed after 8 hr of UV irradiation. The reaction rates of EV dye degradation on P25-TiO_2_ have been prepared to compare with those on ZnO/Zn system without or with UV or visible light irradiation under identical condition (Figure 6). As a comparsion, P-25 TiO_2_ was also performed under identical conditions. All the degradation of EV on ZnO or ZnO/Zn was significantly higher than that on the photocatalyst P25. As indicated by the SEM and the XRD, in the dark controlled experiments, the EV dye may have been degraded by Zn in a reduction reaction resulting in the production of ZnO, which enhanced the photocatalytic reaction under photo-irradiation reaction conditions. ZnO-assisted photocatalytic degradation of the triphenylmethane dye under UV-365 nm and visible light irradiation was reported [3,25]. This study clearly reveals that both Zn and ZnO are capable of degrading EV dye, the efficiency of which is primarily provided by the reduction of Zn; however, under photo-irradiation conditions, the ZnO acts as a photocatalyst, which also makes a significant contribution to the degradation of EV.

### Effect of pH and dye concentration

**Figure 7:**
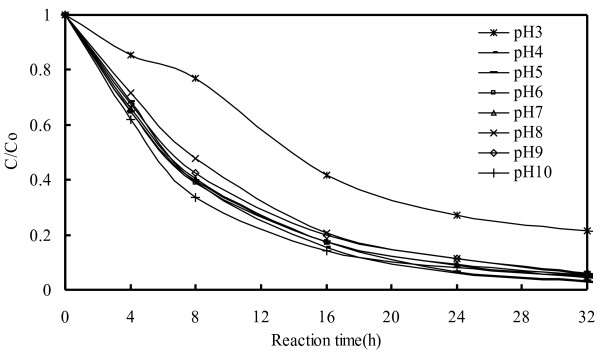
pH effect on the EV degradation rate: Zn foil of Alkaline-Manganese Dioxide-Zinc Cell; EV = 10 ppm; without UV irradiation.

The degradation rate of the EV dye as a function of reaction pH is shown in Figure 7. The degradation rate of the EV dye remained relatively constant as the pH of the solution was increased. A reduction in the degradation rate was clearly observed at pH 3, and might be attributable to the reaction of zinc metal with protons, and the subsequent slow release of hydrogen gas. It has been reported that the Zn metal in acidic water might result in the formation of ZnO film associated with the evolution of hydrogen [31]. In typical textile effluent, dye concentrations range from 150 to 200 ppm. By varying the initial dye concentrations from 10 to 200 ppm at constant Zn-foil loading (pH=6), we determined the effect of dye concentration on the degradation rate, the results of which are shown in Additional file 1: Figure S1. Degradation efficiency is inversely influenced by the concentration of the dye, which can be explained by the fact that as the concentration of the dye increased, the equilibrium adsorption time of dye on the Zn-foil surface active sites increased. Thus, the competitive adsorption of O_2_ on the same sites decreased, resulting in a reduced ZnO formation rate and lower EV dye degradation efficiency.

### Reusability of Zn-foil

**Figure 8:**
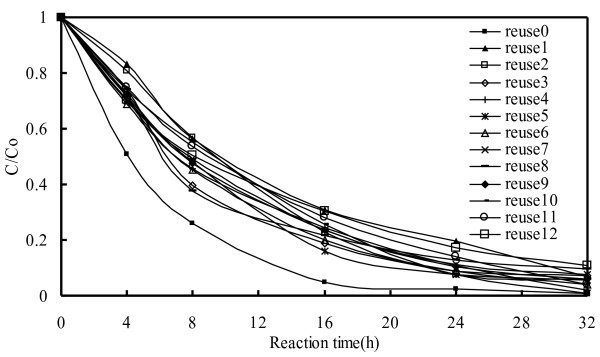
Comparison of degradation rate for the degradation of EV under Zn foil of Alkaline-Manganese Dioxide-Zinc Cell and reused Zn foil (10 ppm, pH = 6).

This experiment involved a comparison of degradation efficiency resulting from the use of freshly prepared Zn-foil compared with that of reused Zn-foil under the same processing conditions. As shown in Figure 8, reused Zn-foil exhibited lower degradation efficiency than fresh Zn-foil, which could be attributed to a reduction in the number of active sites on the surface of the reused Zn-foil. As indicated in Figure 8, degradation efficiency is unaffected by the duration of reactions using reused Zn-foil. This can be explained by the fact that the ZnO is continuously formed and peels off from the Zn-foil surface leaving ZnO powders following the reaction, as depicted in the SEM images in Figure 3 showing irradiation times ranging from 8 hr to 64 hr.

### Separation of the intermediates

**Figure 9:**
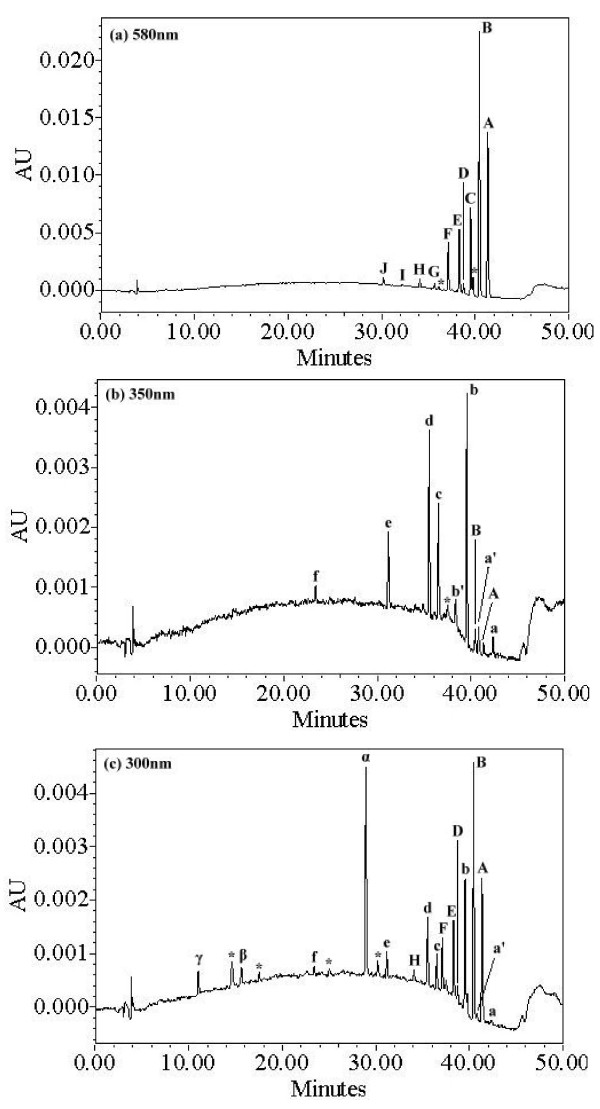
**HPLC chromatogram of intermediates at pH 6 (EV, 0.01 gL**^**-1**^**), 36 h of reaction with Zn-foil, recorded at (a) 580 nm, (b) 350 nm, and (c) 300 nm, impurities were marked with star.**

**Figure 10:**
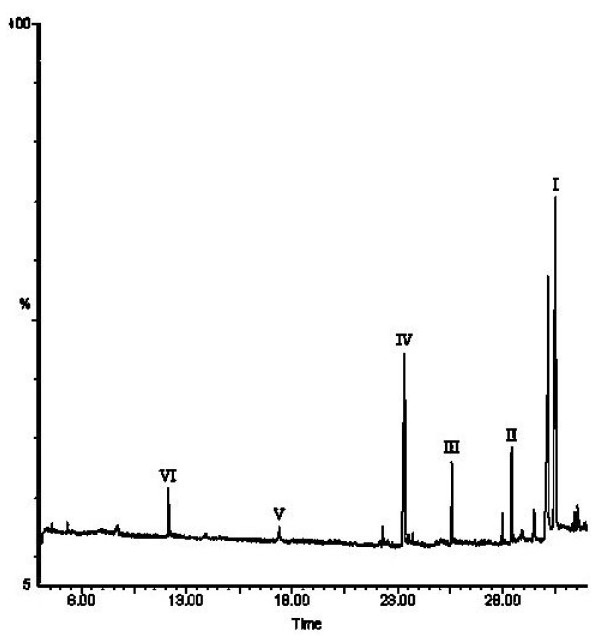
GC-MS/EI chromatogram obtained for a SPE extract of solution after 36 h of EV dye with Zn-foil under dark condition.

Chromatograms recorded at 580, 350, 300 nm are illustrated in Figure 9. With an increase in UV irradiation time to 36 hr at pH 6, twenty-one components were identified within a 50 min retention time. The EV dye and its related intermediates were denoted as species A-J, a-f, a′-b′, and α-γ. Except for the initial EV dye (peak A), the peaks initially increased before subsequently decreasing, indicating the formation and the transformation of intermediates. Figure 10 shows the GC chromatogram obtained from an SPME extract of EV solution after 36 hr of photo-irradiation reaction. Six compounds were detected as possible degradation intermediates. These intermediates were denoted as compounds I-VI.

### UV-visible spectra of the intermediates

The UV-PDA adsorption spectra of the intermediates are depicted in the Additional file 1: Figure S2, identified as A-J and a-f, corresponding to the peaks A-J and a-f, in Figure 9, respectively. The maximum absorption of the spectral band shifted from 591.8 nm (spectrum A) to 561.7 nm (spectrum I), and from 371.6 nm (spectrum a) to 340.6 nm (spectrum f). Presumably, these shifts are due to the formation of a series of *N*-de-ethylated and *N*-hydroxyethylated intermediates. From these results, several groups of intermediates were identified.

The first group is marked in the chromatogram and illustrated in Figure 9(a). The major absorption bands of the intermediates of *N*-de-ethylated EV dye are shifted toward the blue region, λ_max_, A (EV), 591.8 nm; B, 585.6 nm; C, 571.9 nm; D, 573.2 nm; E, 582.1 nm; F, 569.7 nm; G, 565.6 nm; H, 563.6 nm; I, 561.7 nm. The *N*-de-ethylation of the EV dye causes the shift in wavelength because of an attack on the *N, N*-diethyl or *N*-ethyl group by the active oxygen species, as depicted in Additional file 1: Figure S2. It has been reported [35] that EV dye is *N*-de-ethylated in a stepwise manner (i.e., Ethyl groups are removed stepwise as confirmed by the blue shifts in the maximum absorbance of the separated intermediates).

The second and third groups are marked in the chromatogram in Figure 9(b). Destruction of EV yields DAP, DDBP, and *N*-de-ethylated products, *N*-hydroxyethylated intermediates. The *N*-de-ethylation derivatives of the DDBP and the *N*-hydroxyethylated intermediates of the *N*-de-ethylated DDBP species, produced by the cleavage of the EV chromophore ring structure, have their λ_max_ blue shifted: a, 371.6 nm; b, 366.7 nm; c, 365.5 nm; d, 367.9 nm; e, 352.6 nm; f, 340.6 nm. The proposed intermediate a compared well with standard material of 4-(*N*, *N*-diethylamino)-4′-(*N*′, *N*′-diethylamino) benzophenone.

The fourth and the fifth groups are marked in the chromatogram and illustrated in Figure 9(c). The *N*-de-ethylation derivatives of the DAP, produced by the cleavage of the EV chromophore ring structure, also have their λ_max_ blue shifted: α, 290.4 nm; β, 282.1 nm; γ, 272.6 nm as previously reported [36].

### Mass spectra of the intermediates

Intermediates were further identified using HPLC-ESI mass spectrometry, and the relevant mass spectra are illustrated in the Additional file 1: Figure S3 and further summarized in Table 1. The molecular ion peaks of the intermediates all appeared in protonated form. Results confirm that component A (m/z = 456.49) is EV. The other components are B, m/z = 428.88; C, m/z = 400.21; D, m/z = 400.19; E, m/z = 372.16; F, m/z = 372.21; G, m/z = 344.19; a, m/z = 325.45; b, m/z = 297.48; c, m/z = 269.31; d, m/z = 269.45; e, m/z = 241.18; f, m/z = 213.13; α, m/z = 166.25; β, m/z = 137.15; γ, m/z = 109.35. The other intermediates are shown in the GC-MS/EI chromatogram, and the relevant mass spectra are illustrated in the Additional file 1: Figure S4. Table 1 presents the molecular ions of intermediates (I-VI), which characterize its corresponding structure. The peaks eluting at 30.48, 28.49, 25.61, 23.39, 17.57, and 12.38 min during GC-MS were identified as *NN*-diethylaminobenzene, *N*-ethylaminobenzene, aminobenzene, acetamide, 2-propenoic acid, and acetic acid, which matched a standard library search with the values of 87%, 82%, 83%, 71%, 88%, and 95%, respectively (see Additional file 1: Figure S4). The intermediates identified in the study were also reported in a previous study on the MEK/TiO_2_ system [36].

**Table 1:** Intermediates of the degradation of EV identified by HPLC-ESI-MS or GC-EI-MS

**HPLCpeaks**	**Intermediates**	**Abbreviation**	**MS peaks (m/z)**	**Absorption maximum (nm)**
**A**	*N*,*N*,*N*′,*N*′,*N"*,*N"*-hexaethylpararosaniline	EV	456.49	591.8
**B**	*N*,*N*-diethyl-*N*′,*N*′-diethyl-*N"*-ethylpararosaniline	DDEPR	428.88	585.6
**C**	*N*,*N*-diethyl-*N*′-ethyl-*N"*-ethylpararosaniline	DEEPR	400.21	571.9
**D**	*N*,*N*-diethyl-*N*′,*N*′-diethylpararosaniline	DDPR	400.19	573.2
**E**	*N*-ethyl-*N*′-ethyl-*N"*-ethyl pararosaniline	EEEPR	372.16	582.1
**F**	*N*,*N*-diethyl-*N*′-ethylpararosaniline	DEPR	372.21	569.7
**G**	*N*-ethy-*N*′-ethylpararosaniline	EEPR	344.19	565.6
**H**	*N*,*N*-diethylpararosaniline	DPR	N/A	563.6
**I**	*N*-ethylpararosaniline	EPR	N/A	561.7
**J**	pararosaniline	PR	N/A	N/A
**a**	4-(*N*,*N*-diethylamino)-4′-(*N*′,*N*′-diethylamino)benzophenone	DDBP	325.45	371.6
**b**	4-(*N*,*N*-diethylamino)-4′-(*N*′-ethylamino)benzophenone	DEBP	297.48	366.7
**c**	4-(*N*-ethylamino)-4′-(*N*′-ethylamino)benzophenone	EEBP	269.31	365.5
**d**	4-(*N*,*N*-diethylamino)-4′-aminobenzophenone	DBP	269.45	367.9
**e**	4-(*N*-ethylamino)-4′-aminobenzophenone	EBP	241.18	352.6
**f**	4,4′-bis-aminobenzophenone	BP	213.13	340.6
**a**′	4-(*N*,*N*-diethylamino)-4′-(*N*′-hydroxyethyl-*N*′-ethylamino)benzophenone	DHEBP	341.23	371.6
**b**′	4-(*N*-hydroxyethyl-*N*-ethylamino)-4′-(*N*′-ethylamino)benzophenone	HEEBP	313.08	369.1
**α**	4-(*N*,*N*-diethylamino)phenol	DAP	166.25	290.4
**β**	4-(*N*-ethylamino)phenol	EAP	137.15	282.1
**γ**	4-aminophenol	AP	109.35	272.6
**I**	N,N-diethylaminobenzene	DBz	149	310.7
**II**	N-ethylaminobenzene	EBz	121	301.5
**III**	Aminobenzene	ABz	93	282.9
**IV**	Acetamide	AAm	59	N/A
**V**	2-Propenoic	PAc	72	N/A
**VI**	Acetic	AAc	60	N/A

Conditions: Zn foil, 0.01 gL^-1^ EV, reaction 36 h.

### Degradation mechanisms of EV

**Figure 11:**
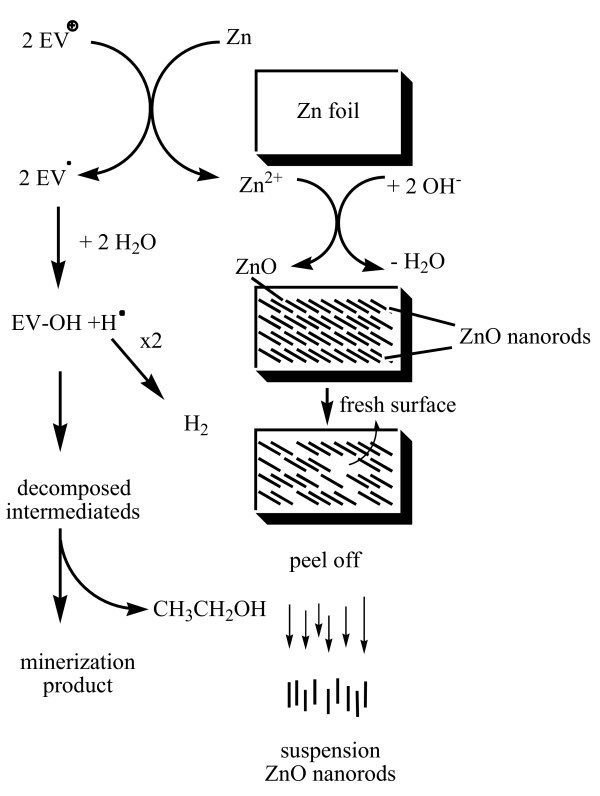
Proposed reaction pathway of EV dye with Zn foil in the aqueous solution.

Most EV^·^ radicals are generated directly from the redox reaction between Zn-foil and surface-adsorbed EV dye. The attack by H_2_O on the ethyl group of diethylamine resulted in *N*-de-ethylated intermediate and ethanol. The above results can be seen more clearly in Figure 11. The degradation reactions occurred at the interface between the Zn metal and liquid water, which resulted in nanostructural ZnO following the evolution of hydrogen [32]. According to earlier reports [3,6], most *N*-de-alkylation processes are preceded by the formation of a nitrogen-centered radical, while the destruction of dye chromophore structures is preceded by the generation of a carbon-centered radical [3,4]. Consistent with this, the degradation of EV must occur via two different photodegradation pathways, namely the destruction of the chromophore structure and *N*-de-ethylation resulting from the two different radicals, either carbon-centered or nitrogen-centered. Undoubtedly, the electrons from the Zn-foil attack dye molecules, yielding a dye radical. Following this step, the radical Dye^·^ undergoes hydrolysis and/or deprotonation, according to the different adsorption modes of EV on the Zn surface. Based on the experimental results mentioned above, we tentatively propose the pathways of degradation depicted in Figure 11.

### *N*-de-ethylation of EV

**Figure 12:**
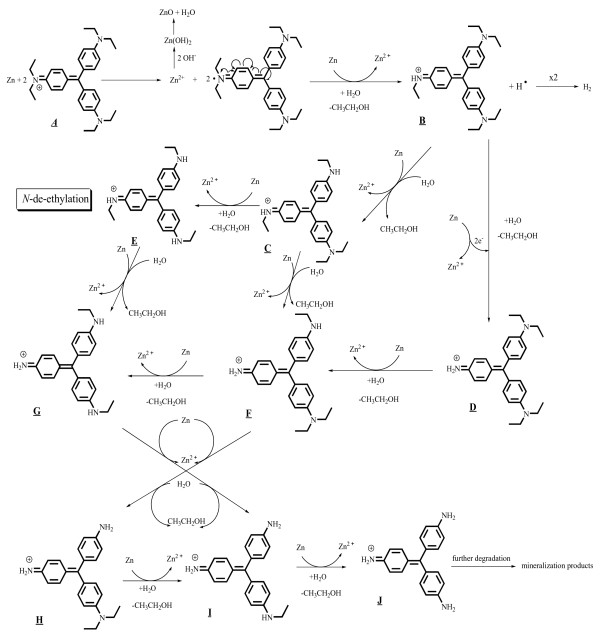
**Proposed *N*-de-ethylation pathway of the EV dye with Zn foil in aqueous solution followed by the identification of several intermediates by HPLC-ESI mass spectral techniques.**

EV receives electrons from the Zn surface via the positive diethylamine group to form EV^·^ radicals and zinc ions. The attack of H_2_O molecules from the ethyl group of diethylamine on the EV^·^ radicals resulted in the formation of *N*-de-ethylated intermediate and ethanol. The mono-de-ethylated dye derivative B can also be adsorbed onto the Zn surface, implicating similar events (electron attack, hydrolysis or deprotonation) to yield the bi-de-ethylated dye derivatives, C and D. The *N*-de-ethylation process described above continues until the formation of the complete de-ethylated dye J. The concentration of the other intermediates may be too low for detection by HPLC-PDA-ESI-MS. The above results can be seen more clearly in Figure 12.

### Destruction of the conjugated structure of the EV

As described above, electrons flow to the EV molecule via the positive diethylamine group. Following the transfer of electrons, the conjugated structure yields a carbon-centered radical, which is subsequently attacked by molecular oxygen, leading ultimately to a and α. The destruction of the conjugated structure of the EV dye most likely occurs through the attack of O_2_ on the carbon-centered radical of the EV, as intermediates a~f are isolated from the HPLC chromatogram. This process also occurs in *N*-de-ethylated EV derivatives (B to F), which are adsorbed on the Zn surface, implicating electrons in other similar events (electron attack, hydrolysis, or deprotonation, and/or oxygen attack) to yield the mono-*N*-de-ethylated derivative b. A similar process occurred in α to produce β. The *N*-de-ethylation process for a and α continues until the formation of the complete *N*-de-ethylated derivative f and γ. All of the above *N*-de-ethylation processes also produced a parallel series of *N*-de-hydroxyethylated intermediates through the hydroxylation of the *N*-ethyl group. All intermediates were further degraded to *N*, *N*-diethylaminobenzene, *N*-ethylaminobenzene, aminobenzene, acetamide, 2-propenoic acid, and acetic acid, which were subsequently mineralized to CO_3_^2–^ and NO_3_^–^[35]. The degradation intermediates clearly reached their maximum concentrations, although some might have been under the detection limit. Mechanisms similar to those proposed here were also observed in a previous study of the MEK/TiO_2_ system [36].

**Figure 13:**
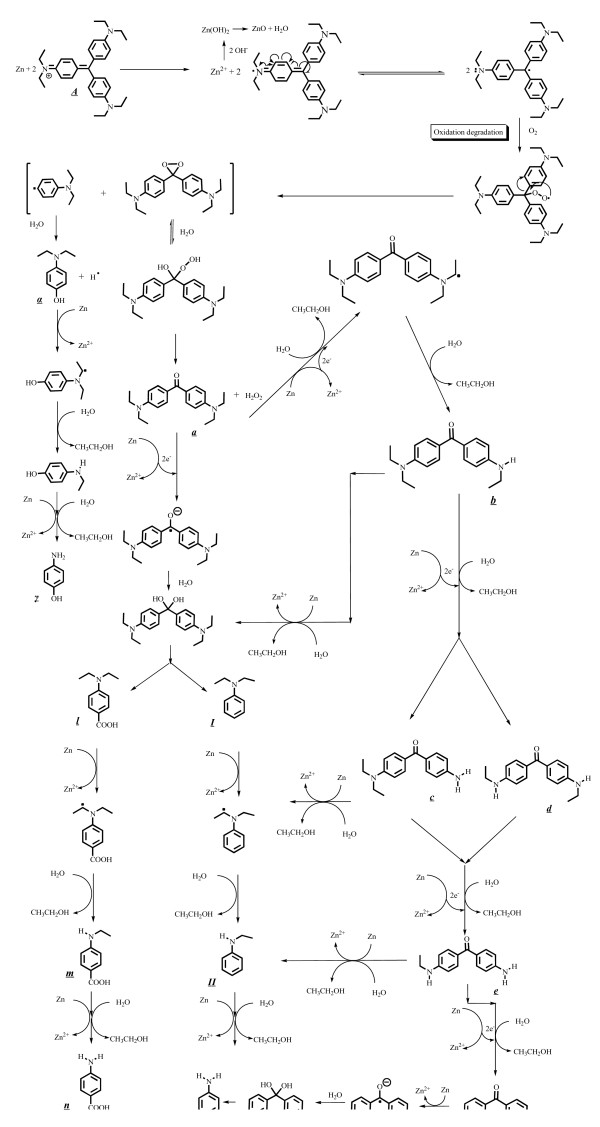
Proposed pathway of the destruction of the conjugated structure of the EV dye with Zn foil followed by the identification of several intermediates by HPLC-ESI mass spectral techniques.

Further evidence related to the pathway(s) of degradation was obtained by GC-MS spectroscopy. From the results of mass spectral analysis, the major components in the gas chromatogram were identified as *N*, *N*-diethylaminobenzene, *N*-ethylaminobenzene, aminobenzene, acetamide, 2-propenoic acid, and acetic acid. The former intermediates (I-III) detected by GC-MS resulted from the cleavage of intermediates in the third group (a-f), leading to aminobenzene derivatives. The latter intermediates (IV-VI) were formed by the cleavage of aromatic derivatives, leading to aliphatic products. The above results can be seen more clearly in Figure 13.

## Conclusions

This paper used HPLC-PDA-ESI-MS analysis to identify the mechanism underlying the degradation of ethyl violet dyes. In this study, a nanostructural ZnO film was produced on the Zn foil from Alkaline-Manganese Dioxide-Zinc cells, providing outstanding potential for future applications in the photocatalytic degradation of organic dye for environmental remediation. This study used HPLC-PDA-ESI-MS and GC-MS to differentiate and characterize twenty-six intermediates of the degradation process. According to variations in the quantity of intermediates, various possible degradation pathways for the decolorization of dyes were also proposed and discussed.

## Competing interests

The author declares that they have no competing interests.

## Authors' contributions

CCC developed the concept, analyzed the data and drafted the manuscript. JXW and WHC carried out the chemical synthesis. WYL and CWW advised on the methods of tests. All authors read and approved the final manuscript.

## Supplementary Material

Additional file 1 **Effect of EV dye concentration, UV-PDA absorption spectra, and mass spectra of intermediates are available for reference.Figure 1S.** UV PDA spectra of intermediates formed during the degradation of EV corresponding to the peaks in the HPLC chromatogram. (a) spectra A-I and (b) spectra a-f corresponded to the peaks A-I denoted in the Figure 10(a) and the peaks a-f denoted in the Figure 10(b) respectively. **Figure 2S.** ESI mass spectra of intermediates formed during the degradation of the EV dye after HPLC separation: mass spectra denoted A-G, a-f, a′-b′ and α-γ corresponded to the A-G, a-f, and α-γ species denoted in the Figure 10 respectively. **Figure 3S.** EI mass spectra of intermediates formed during the degradation of the EV dye after GC separation: mass spectra denoted **I-VI** corresponding to the **I-VI** species in the Figure 11 respectively. **Figure 4S.** EI mass spectra of intermediates formed during the degradation of the EV dye after GC separation: mass spectra denoted **I-VI** corresponding to the **I-VI** species in the **Figure 11** respectively. Click here for file
