# Correction: Determining the degradation efficiency and mechanisms of ethyl violet using HPLC-PDA-ESI-MS and GC-MS

**DOI:** 10.1186/1752-153X-8-24

**Published:** 2014-04-17

**Authors:** Chung-Shin Lu, Wan-Yu Lin, Jian-Xun Wang, Chia-Wei Wu, Chiing-Chang Chen

**Affiliations:** 1 Department of Science Application and Dissemination, National Taichung University of Education, Taichung 403, Taiwan; 2 Department of Plant Pathology, National Chung Hsing University, Taichung 402, Taiwan; 3 Department of General Education, National Taichung, University of Science and Technology, Taichung 403, Taiwan

## Abstract

This is a correction to the following paper: Determining the degradation efficiency and mechanisms of ethyl violet using HPLC-PDA-ESI-MS and GC-MS, Wen-Hsin Chung, Chung-Shin Lu, Wan-Yu Lin, Jian-Xun Wang, Chia-Wei Wu, Chiing-Chang Chen, Chemistry Central Journal 2012, 6:63 (30 June 2012).

## Correction

This is a correction to the following paper: Determining the degradation efficiency and mechanisms of ethyl violet using HPLC-PDA-ESI-MS and GC-MS, Wen-Hsin Chung, Chung-Shin Lu, Wan-Yu Lin, Jian-Xun Wang, Chia-Wei Wu, Chiing-Chang Chen, Chemistry Central Journal 2012, 6:63 (30 June 2012).

Wen-Hsin Chung has requested that his name is removed from the original article [1] because the article was submitted without his permission.
