# Supplementary material for: Determining the degradation efficiency and mechanisms of ethyl violet using HPLC-PDA-ESI-MS and GC-MS
Source: Chem Cent J. 2012 Jun 30;6:63. doi: 10.1186/1752-153X-6-63 (PMC3544139; doi:10.1186/1752-153X-6-63)
Supplement: Additional file 1 — Effect of EV dye concentration, UV-PDA absorption spectra, and mass spectra of intermediates are available for reference.Figure 1S. UV PDA spectra of intermediates formed during the degradation of EV corresponding to the peaks in the HPLC chromatogram. (a) spectra A-I and (b) spectra a-f corresponded to the peaks A-I denoted in the Figure 10(a) and the peaks a-f denoted in the Figure 10(b) respectively. Figure 2S. ESI mass spectra of intermediates formed during the degradation of the EV dye after HPLC separation: mass spectra denoted A-G, a-f, a′-b′ and α-γ corresponded to the A-G, a-f, and α-γ species denoted in the Figure 10 respectively. Figure 3S. EI mass spectra of intermediates formed during the degradation of the EV dye after GC separation: mass spectra denoted I-VI corresponding to the I-VI species in the Figure 11 respectively. Figure 4S. EI mass spectra of intermediates formed during the degradation of the EV dye after GC separation: mass spectra denoted I-VI corresponding to the I-VI species in the Figure 11 respectively. [file 1752-153X-6-63-S1.doc]

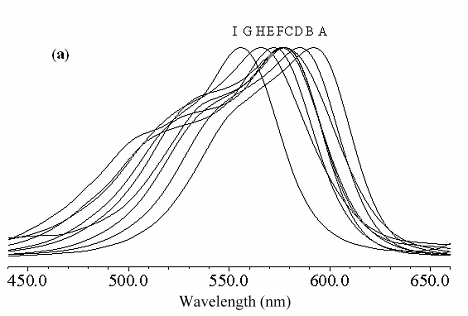


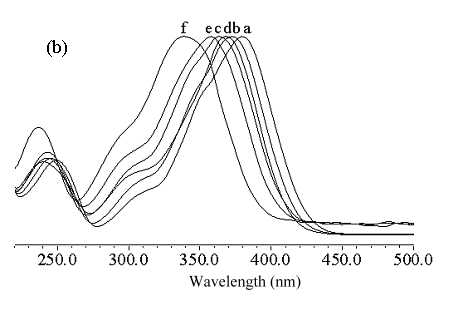


**Figure 1S.** UV PDA spectra of intermediates formed during the degradation of EV corresponding to the peaks in the HPLC chromatogram. (a) spectra A-I and (b) spectra a-f corresponded to the peaks A-I denoted in the **Figure 10(a)** and the peaks a-f denoted in the **Figure10(b)** respectively.


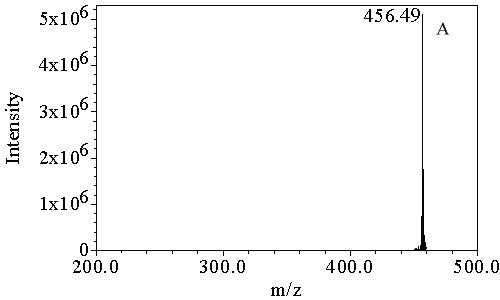

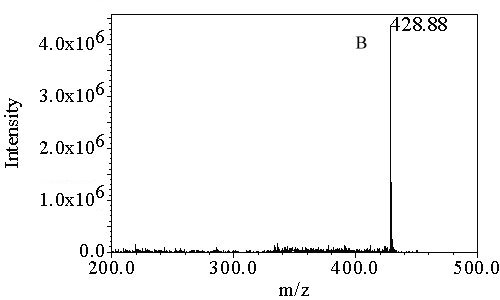

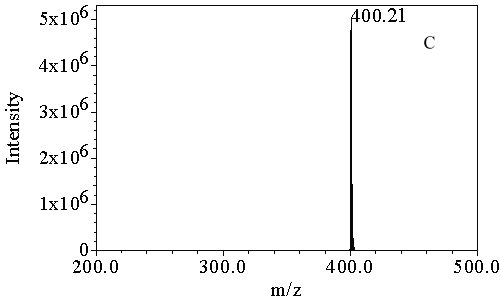

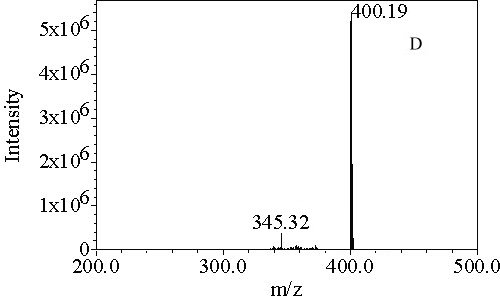

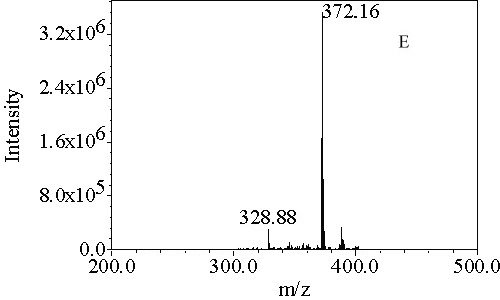

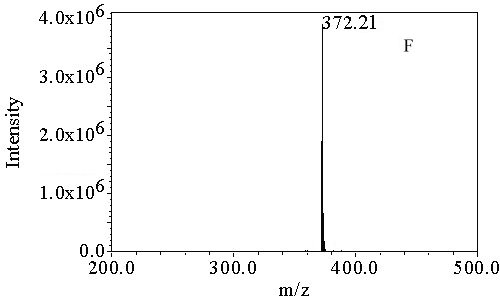

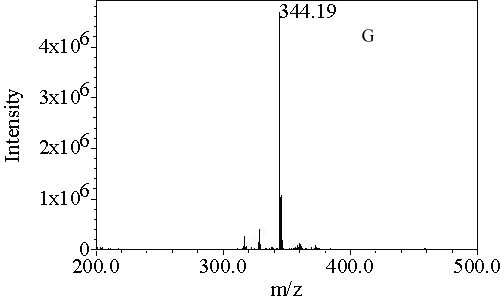

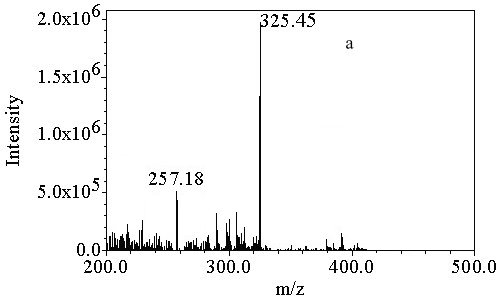

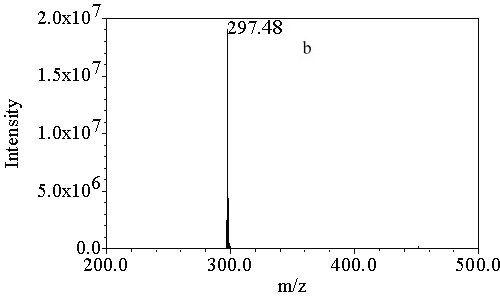

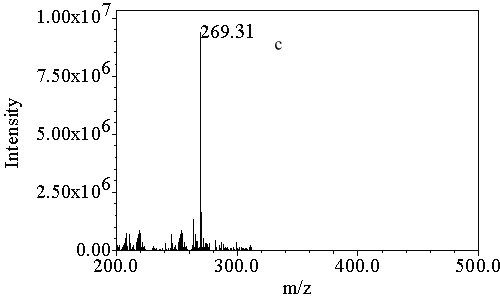

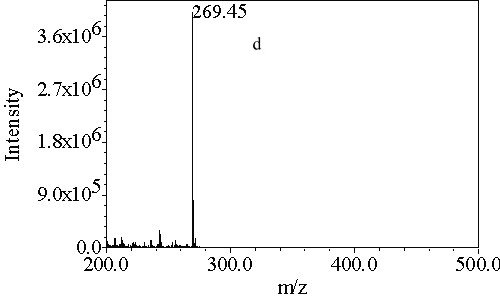

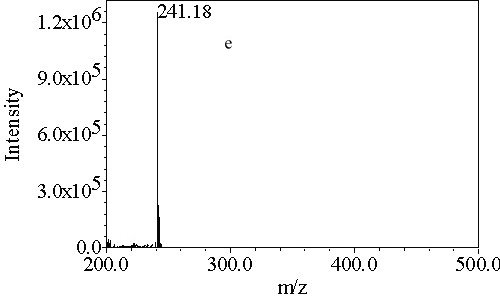

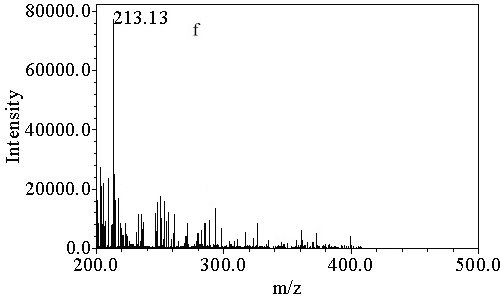

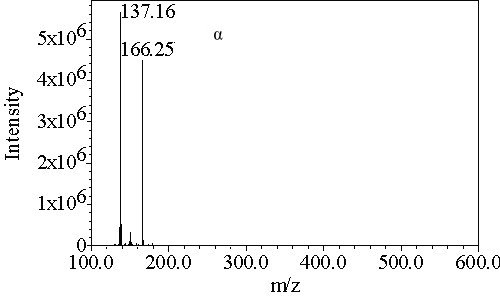

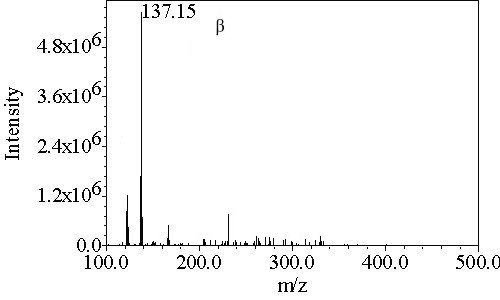

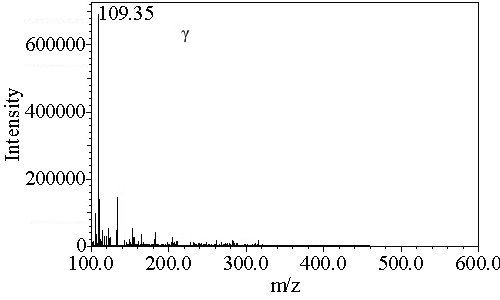


**Figure 2S.** ESI mass spectra of intermediates formed during the degradation of the EV dye after HPLC separation: mass spectra denoted A-G, a-f, a′-b′ and α-γ corresponded to the A-G, a-f, and α-γ species denoted in the **Figure 10** respectively.

| **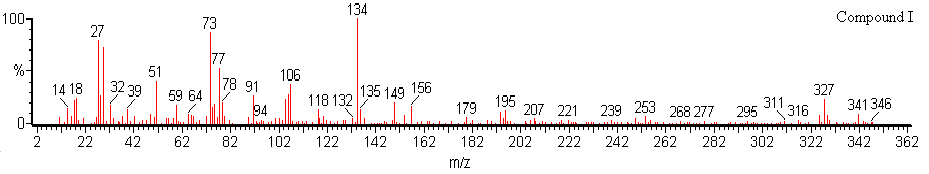** |
| --- |
| 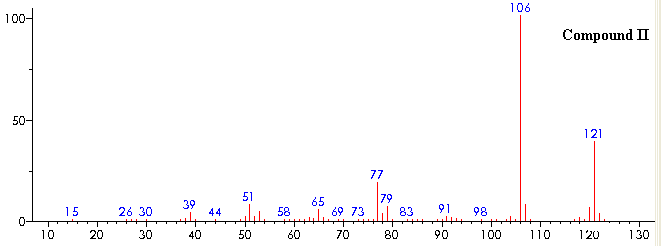 |
| **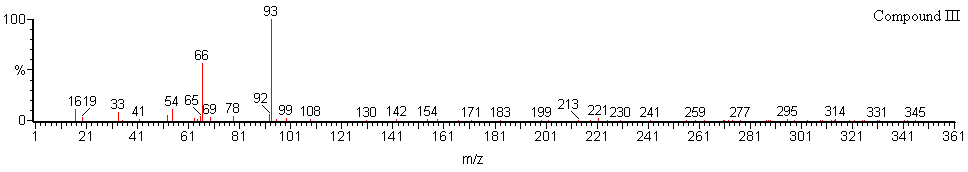** |
| **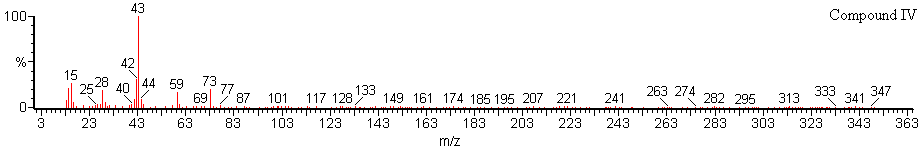** |
| **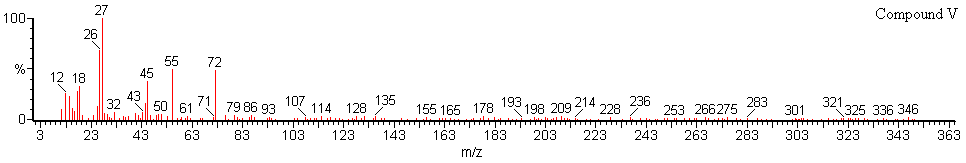** |
| **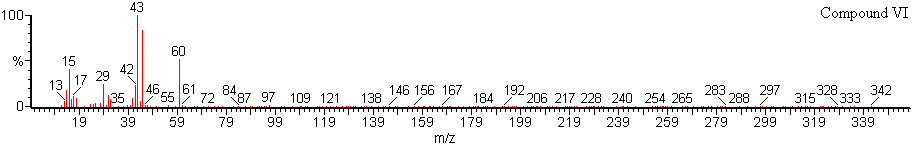** |

**Figure 3S.** EI mass spectra of intermediates formed during the degradation of the EV dye after GC separation: mass spectra denoted **I-VI** corresponding to the **I-VI** species in the **Figure 11** respectively.
